# High-throughput in vitro prediction of available energy in feed ingredients for pigs using a novel computer-controlled digestion system

**DOI:** 10.1093/jas/skag071

**Published:** 2026-03-09

**Authors:** Yuming Wang, Jiangtao Zhao, Jinyuan Zhang, Chenxu Li, Hu Zhang, Feng Zhao, Lixiang Gao, Jingjing Xie

**Affiliations:** State Key Laboratory of Animal Nutrition and Feeding, Institute of Animal Science of Chinese Academy of Agricultural Sciences, Beijing 100193, China; National Reference Laboratory of Veterinary Drug Residues (HZAU) and MAO Key Laboratory for Detection of Veterinary Drug Residues, Huazhong Agricultural University, Wuhan, Hubei 430070, China; Wen’s Food Group Co. Ltd., Yunfu, Guangdong 527439, China; State Key Laboratory of Animal Nutrition and Feeding, Institute of Animal Science of Chinese Academy of Agricultural Sciences, Beijing 100193, China; State Key Laboratory of Animal Nutrition and Feeding, Institute of Animal Science of Chinese Academy of Agricultural Sciences, Beijing 100193, China; State Key Laboratory of Animal Nutrition and Feeding, Institute of Animal Science of Chinese Academy of Agricultural Sciences, Beijing 100193, China; State Key Laboratory of Animal Nutrition and Feeding, Institute of Animal Science of Chinese Academy of Agricultural Sciences, Beijing 100193, China; Wen’s Food Group Co. Ltd., Yunfu, Guangdong 527439, China; State Key Laboratory of Animal Nutrition and Feeding, Institute of Animal Science of Chinese Academy of Agricultural Sciences, Beijing 100193, China

**Keywords:** additivity, available energy, growing pig, in vitro digestion, prediction model

## Abstract

This study aimed to establish predictive equations for digestible energy (DE) and metabolizable energy (ME) of feed ingredients using a novel computer-controlled simulated digestion system (CCSDS) and validate the additivity of predicted energy values in complete diets for growing pigs. An *in vivo* experiment was conducted to determine the DE and ME of 30 experimental diets. A total of 60 barrows (initial BW of 37.3 ± 4.7 kg) were used, divided into two replicate blocks of 30 pigs each. Within each block, a 30 × 3 Youden square design was implemented across three experimental periods. Two pigs were allocated per diet during each period, resulting in six replicates per diet. Experimental diets included 20 feed ingredients, and 10 validation diets were formulated with the above feed ingredients to assess energy additivity and accuracy. The in vitro digestible energy (IVDE) was determined using CCSDS with five replicates for each diet. Strong correlations were observed between IVDE and in vivo DE (DE = 1.001 × IVDE + 180, *R^2^* = 0.85, RSD = 310 kcal/kg of DM, *P *< 0.01) and ME (ME = 1.015 × IVDE—29, *R^2^* = 0.89, RSD = 254 kcal/kg of DM, *P *< 0.01), with predicted values closely aligning with determined values across all feed ingredients. The mean IVDE: DE and IVDE: ME were approximately 0.95 and 0.99, respectively, highlighting the high predictive accuracy of CCSDS. Validation diets demonstrated consistent energy additivity. Single sample *t*-tests revealed no difference was observed between predicted and determined DE values in eight out of ten diets, and between predicted and determined ME values in 9 out of 10 diets. In conclusion, these findings underscore the utility of CCSDS as a cost-effective and reliable alternative to in vivo methods, offering significant potential for precise energy assessment in swine feed formulation.

## Teaser

A novel computer-controlled digestion system accurately predicts the available energy content of pig feed, providing a rapid and cost-effective alternative to *in vivo* experiments. This advance facilitates the formulation of more precise and efficient diets for growing pigs, ultimately enhancing production efficiency.

## Introduction

Accurate assessment of the nutritional quality of feed components, particularly their energy bioavailability, is fundamental for optimizing animal production (Costa et al. 2024; Kim et al. 2024). While in vivo animal metabolism trials remain the reference standard for evaluating nutrient digestibility, their practical application is significantly constrained by time, labor intensity, and high costs (Zaefarian et al. 2021; Noblet et al. 2022). Consequently, there is a pressing need for robust in vitro methodologies capable of rapidly and efficiently predicting the nutritional value of complex matrices, including diverse food ingredients and monogastric animal feeds, under controlled laboratory conditions.

For decades, researchers have pursued in vitro digestion approaches that simulate the gastrointestinal digestion and nutrient absorption processes (Johnson 1966). Enzymatically driven in vitro digestion protocols have gained prominence as standardized tools for assessing nutrient release (Sommerfeld and Santos 2021; Son et al. 2024). A significant advancement was the three-step method developed by Boisen and Fernández (1997), simulating digestion in the stomach, small intestine, and large intestine of growing pigs. While this approach demonstrated a strong correlation (*r *= 0.93) between in vitro and in vivo energy digestibility across various samples, it exhibited an average absolute difference of 7.1%. Subsequent studies reported even larger discrepancies (>9.0%) for conventional feed ingredients (Woyengo et al. 2016; Choi et al. 2021). Additionally, the accuracy of such manual in vitro methods is highly dependent on sample characteristics. For instance, weak correlations (*r *= 0.14) were observed between in vitro and in vivo dry matter (DM) digestibility in specific sample sets like distillers dried grains with solubles (DDGS; Huang et al., 2017a, 2018a). Key limitations contributing to these inconsistencies include: 1) enzyme concentrations often based on weight-to-volume ratios rather than physiologically relevant activity levels (Boisen and Fernández 1997); 2) oversimplified enzyme cocktails for large intestine simulation (eg, reliance on Viscozyme, a multi-enzyme complex containing six cellulases), neglecting the significant roles of proteases and amylases (Du et al. 2023); and 3) inherent variability associated with manual procedures (Kim et al. 2024).

To overcome these challenges, we recently developed a novel computer-controlled simulated digestion system (CCSDS; Du et al. 2022, 2023; Gao et al. 2023). This system precisely regulates digestion time, digestive fluid composition, and enzyme activity levels to better align with in vivo physiological conditions. Initial validation demonstrated the CCSDS’s effectiveness in accurately predicting digestible energy (DE) for unconventional plant protein meals (in vitro digestible energy [IVDE]:DE = 0.96, *r *= 0.95, n = 9) and cereals (IVDE: DE = 1.01, *r *= 0.84, n = 14) (Du et al., 2022; Gao et al., 2023). We hypothesize that this advanced in vitro platform can provide accurate predictions of both DE and metabolizable energy (ME) across a wide range of feed ingredients, including cereals, plant protein sources, and fibrous materials. Moreover, we propose that these predicted energy values possess additive properties within complete formulations.

Therefore, the objectives of this study were to: 1) establish robust predictive equations for DE and ME of diverse ingredients using the CCSDS, and 2) validate the additivity of these predicted values in complete diets. This work aims to provide a reliable, high-throughput analytical tool for the precise assessment of energy bioavailability in complex feed matrices.

## Materials and methods

### IACUC statement

All animal procedures in this study were reviewed and approved by the Animal Care and Welfare Committee of the Institute of Animal Science of Chinese Academy of Agricultural Sciences, with the approval number IAS2021-60. This study was conducted in strict accordance with the Chinese National Standard GB/T 35892-2018 (Laboratory Animal-Guideline for ethical review of animal welfare).

### Feed ingredients and experimental diets

A total of 20 commonly used feed ingredients for growing pigs in China were selected for this experiment (Table 1, Wen’s Food Group Co. Ltd., Guangdong, China), including six cereal grains: corn, wheat, sorghum, barley, wheat flour, and brown rice; 11 protein feed ingredients: wheat middling, soybean meal 1 (43% crude protein [CP]), soybean meal 2 (46% CP), fermented soybean meal, enzyme-hydrolyzed soybean meal, extruded soybean, soy protein concentrate, cottonseed meal, rapeseed meal, sunflower meal, and rice DDGS; and three brans: rice bran, defatted rice bran, and wheat bran.

**Table 1:** Chemical composition of feed ingredients (%, DM basis).

Items	DM	GE, kcal/kg	CP	EE	ash	NDF	ADF
**Corn**	88.12	4,459	9.60	4.11	1.68	10.23	1.98
**Wheat**	90.40	4,422	14.62	2.05	1.64	12.39	2.96
**Sorghum**	88.20	4,470	10.48	2.36	1.65	9.46	2.62
**Barley**	89.91	4,354	10.86	2.89	2.66	19.01	6.07
**Wheat flour**	87.08	4,418	14.15	1.97	1.33	7.58	0.69
**Brown rice**	86.81	4,377	9.79	2.33	1.57	4.55	1.24
**Wheat middling**	88.64	4,553	20.48	2.97	3.38	21.09	5.49
**Soybean meal 1**	88.15	4,684	50.84	1.82	6.68	12.60	7.04
**Soybean meal 2**	87.14	4,655	54.63	1.02	7.15	10.93	5.70
**Fermented soybean meal**	93.05	4,744	57.36	1.66	7.22	22.47	8.41
**Enzyme-hydrolyzed soybean meal**	89.46	4,812	61.49	1.42	8.10	17.65	3.72
**Extruded soybean**	91.96	5,750	40.13	23.23	5.17	13.98	6.19
**Soy protein concentrate**	91.43	4,885	72.06	1.29	6.92	24.19	4.63
**Cottonseed meal**	89.89	4,647	51.35	0.65	6.97	31.32	16.17
**Rapeseed meal**	88.22	4,724	42.97	1.96	7.27	28.37	19.82
**Sunflower meal**	90.13	4,650	42.00	2.33	6.91	30.96	19.87
**Rice DDGS**	92.87	4,950	25.32	8.57	9.82	65.13	38.75
**Rice bran**	90.45	5,040	14.00	20.08	7.59	14.09	5.09
**Defatted rice bran**	89.76	4,150	17.50	1.18	9.42	20.55	9.81
**Wheat bran**	90.06	4,584	18.62	2.55	5.48	41.58	12.97
**Mean**	89.59	4,666	31.91	4.32	5.43	20.91	8.96
**Coefficient of variation**	2.03	7.20	64.55	142.85	51.96	66.76	100.79

ADF, acid detergent fiber; CP, crude protein; DDGS, distillers dried grains with solubles; DM, dry matter; EE, ether extract; GE, gross energy; NDF, neutral detergent fiber.

To determine the available energy (DE and ME) of test ingredients, two types of experimental diets were formulated (Table 2). For the six cereal grains, diets consisted primarily of the test grain supplemented with necessary minerals and vitamins. For the remaining 14 ingredients (eg, protein ingredients, processed by-products), a corn-based basal diet was used, in which corn was partially replaced by the test ingredient to maintain a similar energy density and evaluate the ingredient’s incremental energy contribution. Additionally, 10 validation diets were formulated by combining 3 to 6 test ingredients (Table 3), which was selected to realistically simulate the major ingredients commonly used in commercial swine feed formulation, thereby testing the practical applicability of the energy additivity principle. All diets were supplemented with dicalcium phosphate, limestone, salt, and a vitamin-mineral premix to meet nutritional requirements. In total, 30 experimental diets were used in this study.

**Table 2:** The composition of experimental diets (%, DM basis).

Items	Experimental diets																			
	1	2	3	4	5	6	7	10	11	12	13	14	15	16	17	18	19	8	9	20
**Corn**	96.96						37.07	67.86	68.06	67.17	70.41	71.38	76.45	76.88	72.6	71.9	71.22	66.71	66.71	71.94
**Wheat**		97.58																		
**Sorghum**			97.37																	
**Barley**				97.24																
**Wheat flour**					97.19															
**Brown rice**						96.86														
**Wheat middling**							60.06													
**Soybean meal 1**								29.44												
**Soybean meal 2**									29.24											
**Fermented soybean meal**										30.28										
**Enzyme-hydrolyzed soybean meal**											26.89									
**Extruded soybean**												25.87								
**Soy protein concentrate**													20.8							
**Cottonseed meal**														20.33						
**Rapeseed meal**															25					
**Sunflower meal**																25.46				
**Rice DDGS**																	25.97			
**Rice bran**																		30.36		
**Defatted rice bran**																			30.48	
**Wheat bran**																				25.19
**Dicalcium phosphate**	1.23	0.88	1.01	1.14	1.09	1.37	1.07	1.06	1.06	1.04	1.05	1.18	1.05	0.9	0.85	0.98	1.12	1.06	0.96	0.91
**Limestone**	1.21	0.95	1.02	1.03	1.12	1.17	1.2	1.04	1.04	0.92	1.06	0.98	1.11	1.29	0.95	1.06	1.1	1.27	1.25	1.36
**Salt**	0.46	0.45	0.46	0.45	0.46	0.46	0.46	0.46	0.46	0.45	0.45	0.45	0.45	0.46	0.46	0.46	0.45	0.46	0.46	0.46
**Premix^1^**	0.14	0.14	0.14	0.14	0.14	0.14	0.14	0.14	0.14	0.14	0.14	0.14	0.14	0.14	0.14	0.15	0.14	0.14	0.14	0.14
**Total**	100.00	100.00	100.00	100.00	100.00	100.00	100.00	100.00	100.00	100.00	100.00	100.00	100.00	100.00	100.00	100.00	100.00	100.00	100.00	100.00
**Analyzed chemical composition**																				
**DM**	88.00	89.71	88.09	89.74	87.96	87.79	88.65	89.53	89.31	88.29	87.89	89.81	89.46	89.69	88.62	88.75	88.32	88.88	89.65	89.11
**GE, kcal/kg**	4,327	4,325	4,358	4,234	4,308	4,253	4,401	4,490	4,239	4,414	4,396	4,432	4,435	4,657	4,430	4,377	4,417	4,397	4,467	4,349
**CP**	9.20	14.79	9.99	10.43	13.58	9.62	15.44	10.31	11.19	21.64	22.06	23.71	22.86	16.87	21.44	17.35	17.29	16.31	12.78	11.38
**ash**	4.13	3.61	3.63	4.88	3.76	4.02	4.91	5.86	6.35	5.23	5.30	5.28	5.57	4.91	5.04	5.16	4.89	5.23	6.01	4.89
**NDF**	8.58	9.81	9.25	18.32	6.94	3.15	15.65	10.92	12.20	10.05	9.25	13.33	10.72	8.97	11.80	11.29	13.63	13.13	23.53	16.60
**ADF**	1.95	2.72	2.62	6.49	0.34	0.95	3.97	3.10	4.42	3.54	3.03	3.78	2.41	3.24	4.24	4.62	6.20	6.22	11.96	4.78

ADF, acid detergent fiber; CP, crude protein; DDGS, distillers dried grains with solubles; DM, dry matter; GE, gross energy; NDF, neutral detergent fiber.

1 The premix provided the following per kilogram of diets (as-fed basis): vitamin A 6,400 IU, vitamin D_3_ 2,200 IU, vitamin E 50 mg, vitamin K_3_ 2 mg, vitamin B_1_ 2 mg, vitamin B_2_ 5 mg, vitamin B_6_ 3 mg, vitamin B_12_ 24 μg, pantothenic acid 12 mg, nicotinic acid 20 mg, folic acid 1 mg, biotin 0.12 mg, Cu (as copper sulfate) 10 mg, Fe (as ferrous sulfate) 110 mg, Zn (as zine sulfate) 40 mg, Mn (as manganese sulfate) 25 mg, Se (as sodium selenite) 0.3 mg, I (as potassium iodide) 0.3 mg. The same as Table 3.

**Table 3:** The composition of the validation diets (%, DM basis).

Items	Validation diets									
	1	2	3	4	5	6	7	8	9	10
**Corn**	62.44	32.20	31.13	32.11	34.82	46.15	57.78	61.44	61.24	62.85
**Wheat**		20.36		10.18		10.02				
**Sorghum**			19.92	9.96						
**Barley**			20.24		10.11					
**Wheat flour**					9.94	9.80				
**Brown rice**		19.75		9.87						
**Wheat middling**					10.01					
**Soybean meal 1**	26.81	25.05	26.12	25.13	22.32				8.05	10.88
**Fermented soybean meal**						21.55				
**Extruded soybean**										10.38
**Soy protein concentrate**								5.21		5.21
**Cottonseed meal**							15.13		10.18	
**Rapeseed meal**								19.83	9.97	
**Sunflower meal**							14.42			
**Rice DDGS**								11.17		
**Rice bran**				10.14		9.99				
**Defatted rice bran**					10.18		10.14			
**Wheat bran**	8.08								8.06	8.02
**Dicalcium phosphate**	0.97	1.03	1.01	0.97	0.95	0.95	0.75	0.83	0.76	0.99
**Limestone**	1.10	1.01	0.98	1.04	1.07	0.95	1.19	0.93	1.14	1.08
**Salt**	0.46	0.46	0.46	0.46	0.46	0.45	0.45	0.45	0.46	0.45
**Premix**	0.14	0.14	0.14	0.14	0.14	0.14	0.14	0.14	0.14	0.14
**Total**	100.00	100.00	100.00	100.00	100.00	100.00	100.00	100.00	100.00	100.00
**Analyzed chemical compositions**										
**DM**	88.09	88.08	88.14	88.14	88.41	89.47	88.69	88.72	88.33	88.90
**GE, kcal/kg**	4,428	4,391	4,407	4,460	4,373	4,459	4,376	4,491	4,434	4,561
**CP**	20.78	20.42	20.26	20.68	20.95	21.08	20.76	20.81	20.73	20.69
**ash**	5.40	5.07	5.18	5.47	5.84	5.45	5.99	5.72	5.40	5.15
**NDF**	11.84	8.66	11.80	9.68	12.37	10.18	13.99	18.50	14.73	12.12
**ADF**	3.97	3.10	4.46	3.61	4.59	3.42	6.78	9.37	6.19	3.98

ADF, acid detergent fiber; CP, crude protein; DDGS, distillers dried grains with solubles; DM, dry matter; GE, gross energy; NDF, neutral detergent fiber.

### In vivo management and sampling

Due to facility constraints (30 metabolism pens available), the in vivo experiment was conducted in two sequential batches. Each batch involved 30 crossbred barrows (Duroc × Landrace × Yorkshire) with an initial body weight (BW) of 37.3 ± 4.7 kg and followed a 30 × 3 Youden square design across three periods. Within each batch and period, each of the 30 experimental diets was assigned to one pig. Consequently, each diet accumulated three replicates per batch (one replicate per period), and a total of six replicates per diet across the two complete batches. Pigs were removed after three periods as their BW approached approximately 80 kg, a range within which nutrient digestibility is considered stable in growing pigs. No pig received the same diet more than once during study. All pigs were individually housed in metabolism pens equipped with slatted floors, a feeder, and a nipple drinker in an environmentally controlled room, where the temperature was maintained at 23 ± 2 °C.

Each period includes a 5-d adaptation period to the experimental diet, followed by a 5-d total collection of feces and urine. Pigs had ad libitum access to water, while average daily feed intake was 4.0% of the initial BW in each period, divided into two equal meals and fed at 0800 and 1600 h daily. Feed refusals and spillage were collected after each meal, dried, weighed, and recorded. Feces were collected using a marker-to-marker (ferric oxide) approach by adding 1 g of ferric oxide to 100 g of the diet at 0800 h on d 6 and 11. Feces were collected between the first and second appearances of red in the feces and were stored at −20 °C immediately after collection (Adeola and Kong 2014; Kong and Adeola 2014). Due to the diverse physical and chemical properties of the test ingredients, the total gastrointestinal transit time varied among diets. Consequently, the fecal collection period varied between 5 and 8 d. Urine was collected daily in a bucket containing 50 mL of 6 mol/L HCl, covered with gauze to prevent contamination from solids. Urine was collected over 5 d, from 0800 h on d 6 to 0800 h on d 11. The urine weight was recorded daily, and 10% of the daily urine weight was transferred to a screw-capped bottle and stored at −20 °C. At the end of the experiment, feces and urine were thawed, pooled separately for each pig and period, and thoroughly mixed to obtain a homogeneous sample. All fecal samples were dried in a forced-air oven at 65 °C for 72 h and subsequently cooled at room temperature for 24 h. The samples were then weighed and subsampled.

### In vitro digestible energy determined with CCSDS

**Figure 1:**
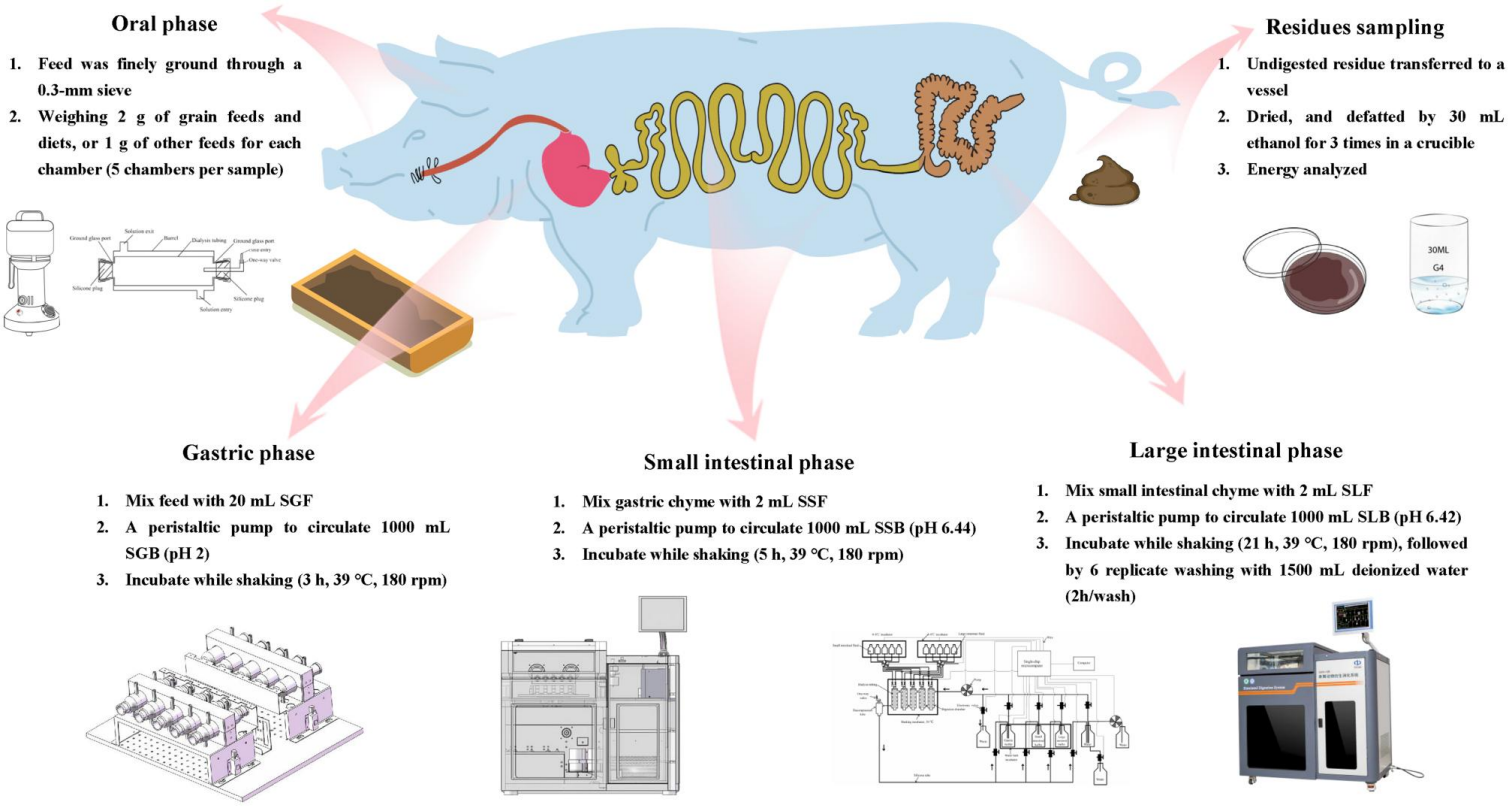
Experimental process of in vitro biomimetic digestion using a novel computer-controlled simulated digestion system. SGB, simulated gastric buffer; SGF, simulated gastric fluid; SLB, simulated large intestine buffer; SLF, simulated large intestine fluid; SSB, simulated small intestine buffer; SSF, simulated small intestine fluid.

The in vitro digestion process was performed following the procedure described by Du et al. (2022) and Gao et al. (2023), as presented in Figure 1. Briefly, each feed sample was ground through a 0.3-mm screen and analyzed in five replicates, with one tube per replicate. Each tube contained 2 g of cereal grain or diet, or 1 g of other feed ingredients. The digestive parameters were set in the computer to automatically control the in vitro digestion process for growing pigs: 3 h for gastric digestion, 5 h for small intestinal digestion, 21 h for large intestinal digestion, and six washing cycles for digestive residues. The buffer solution and reactor cabinet were maintained at 39 °C, with a shaking speed of 180 rpm. After digestion in the CCSDS, the undigested residue in each tube was transferred to a pre-weighed glass vessel and dried overnight at 65 °C and subsequently dried at 105 °C to a constant weight. The dried residue was then transferred to a pre-weighted fritted glass filter crucible (G4) for fat extraction, using 45 mL of ethanol (≥99.7%) for four repetitions. Finally, the crucible was dried at 105 °C for 5 h to a constant weight. Simultaneously, the residual energy in a blank sample (using only digestive fluid and buffer solution) was determined to correct the IVDE value.

Simulated gastrointestinal fluids and buffer solutions were prepared using a commercial assay kit (catalog code IVDEGP, Hunan Zhongben Intelligent Technology Development Co., Ltd., Changsha, China). The simulated gastric fluid consisted of 890 U/mL of pepsin, 80.6 mmol/L of Na^+^, 6.0 mmol/L of K^+^, 134.2 mmol/L of Cl^–^, and a pH of 2.0, maintained at 39 °C. The simulated small intestinal fluid was composed of 4,239 U/mL of amylase, 1,323 U/mL of trypsin, 166 U/mL of chymotrypsin, 89.9 mmol/L of Na^+^, 15.0 mmol/L of K^+^, 116.7 mmol/L of Cl^–^, and a pH of 6.44, also maintained at 39 °C. The simulated large intestinal fluid contained 1,572 U/mL of amylase, 491 U/mL of trypsin, 62 U/mL of chymotrypsin, 0.77 U/mL of cellulase, 93.2 mmol/L of Na^+^, 11.1 mmol/L of K^+^, 25.1 mmol/L of Cl^–^, and a pH of 6.42, maintained at 39 °C.

### Chemical analysis

All samples were finely ground through a 0.42-mm screen. The feed samples were analyzed for DM (method 930.15), CP (method 990.03), ether extract (EE; method 996.01), and ash content (method 942.05) according to AOAC International (2006). The neutral detergent fiber (NDF) and acid detergent fiber (ADF) contents of the feed ingredients were determined using ANKOM F57 filter bags, following the procedure outlined by van Soest et al. (1991). Gross energy (GE) content of feed samples, feces, urine, and *in vitro* residue samples was determined using an isoperibol bomb calorimeter (Model 6400, Parr Instruments, Moline, IL), with benzoic acid used as the calibration standard.

### Calculation

The DE and ME for each experimental diet were calculated using equations 1 and 2, respectively, as described by Wang et al. (2020):


(1) $${\text{DE}}_{d}\left(\text{kcal}/\text{kg}\right)=\left(\text{GEI}\;–\;\text{GEO}\right)/\text{DMI}$$

(2) $${\text{ME}}_{d}\left(\text{kcal}/\text{kg}\right)=\left(\text{GEI}\;–\;\text{GEO}\;–\;\text{GEU}\right)/\text{DMI}$$

In this context, DE_d_ and ME_d_ represent the DE (kcal/kg) and ME (kcal/kg) in the experimental diet of DM, respectively; GEI represents the GE intake (kcal) in the diet, GEO denotes the GE output (kcal) in the feces, GEU refers to the GE output (kcal) in the urine, and DMI indicates the feed intake (kg) of DM.

The DE and ME of corn, wheat, sorghum, barley, wheat flour, and brown rice were calculated using equation 3, while the DE and ME of other feed samples were calculated using equations 4 and 5 (Kong and Adeola 2014):


(3) $${\text{DE}}_{\text{ti}}{\;\text{or}\;\text{ME}}_{\text{ti}}\left(\text{kcal}/\text{kg}\right)={\text{GE}}_{\text{ti}}\times \left(\left({\text{DE}}_{d}{\;\text{or}\;\text{ME}}_{d}\right)/{\text{GE}}_{d}\right)$$

(4) $$D_{\text{ti}}=D_{\text{cd}}+\left(D_{\text{td}}-D_{\text{cd}}\right)/P_{\text{ti}}$$

(5) $${\text{DE}}_{\text{ti}}{\;\text{or}\;\text{ME}}_{\text{ti}}\left(\text{kcal}/\text{kg}\right)={\text{GE}}_{\text{ti}}\times D_{\text{ti}}$$

In which, GE_ti_ and GE_d_ represent the GE (kcal/kg) in the feed ingredient and experimental diet of DM, respectively; DE_ti_ and ME_ti_ denote the DE (kcal/kg) and ME (kcal/kg) in the test feed ingredient of DM, respectively; D_ti_, D_cd_ and D_td_ are the coefficients of digestibility (DE: GE) or metabolizability (ME: GE) of the energy in the test feed ingredient, corn-basal diet, and experimental diet, respectively; P_ti_ is the proportion of energy in the experimental diet contributed by the test feed ingredient.

The IVDE of feed ingredient or experimental diet was calculated using equation 6:


(6) $$\text{IVDE}\;\left(\text{kcal}/\text{kg}\right)=\left(\left({\text{GE}}_{t}-{\;\text{GE}}_{r}\right)+{\;\text{GE}}_{e}\right)/W_{t}$$

In which, GE_t_ represents the GE added (kcal) from the feed ingredient or diet in each digestion chamber, GE_r_ denotes the sum of the GE output (kcal) of the defatted residue collected from each digestion chamber, GE_e_ refers to the GE output (kcal) of the dry residue of digestive enzymes in each digestion chamber, and W_t_ indicates the DM weight (kg) of the feed ingredient or diet added to each digestion chamber.

### Statistical analysis

The average relative difference (ARD) between the determined and predicted available energy of 10 validation diets were calculated according to the following equation:


$$\mathrm{ARD}\;=\;\frac{\sqrt[{2}]{\sum \frac{{\left(\mathrm{Determined}-\mathrm{Predicted}\right)}^{2}}{n}}}{\mathrm{Mean}\;\mathrm{of}\;\mathrm{the}\;\mathrm{determined}}\times 100\%$$

Treatment means for DE, ME, and IVDE were calculated using the MEANS procedure of SAS 9.4 (SAS Inst. Inc., Cary, NC). Outliers were identified based on values outside the range of Q1—1.5 × IQR to Q3 + 1.5 × IQR, using the UNIVARIATE procedure. Correlations between *in vitro* and *in vivo* values were assessed using the CORR procedure. Prediction models for DE or ME based on IVDE were developed using the REG procedure. Statistical significance was determined at *P *< 0.05, and the *R^2^* value was used to evaluate the quality of the prediction model. Significant differences in the intercepts or slopes of the regression models between the determined and calculated or predicted values for the 10 validation diets were tested against 1 or 0 using the MIXED procedure, as described by Kaps and Lamberson (2017).

## Results

### Chemical composition of feed ingredients for growing pigs

There was considerable variation in the analyzed chemical compositions among the 20 feed ingredients (Table 1). The mean concentration of GE, CP, EE, ash, NDF, and ADF were 4,666 kcal/kg (ranging from 4,150 to 5,750 kcal/kg), 31.91% (ranging from 9.60% to 72.06%), 4.32% (ranging from 0.65% to 23.23%), 5.43% (ranging from 1.33% to 9.82%), 20.91% (ranging from 4.55% to 65.13%), and 8.96% (ranging from 0.69% to 38.75%) on a DM basis, respectively. The coefficient of variation (CV) of EE and NDF content all exceeded 100%.

### Energetic values of 30 experimental diets

The energy utilization data for experimental diets is presented in Table 4. DM intake ranged from 1.181 to 1.448 kg/d across the 30 diets. Correspondingly, GE intake varied between 5,089 and 6,710 kcal/d of DM. Fecal energy output exhibited considerable variation, from a minimum of 301 kcal/d to a maximum of 1,550 kcal/d, reflecting the diverse digestibility of the dietary components. Urinary energy output ranged from 55 to 217 kcal/d of DM. These direct measurements of GE intake, fecal loss, and urinary loss provided the essential data for the precise calculation of the in vivo DE and ME of each experimental diet.

**Table 4:** Gross energy intake and excretion of growing pigs fed with different diets (kcal/d, DM basis).

Experimental diets	DMI, kg/d	GE intake	Fecal output	Urinary output
**1**	1.356	5,867	626	123
**2**	1.384	5,986	761	102
**3**	1.214	5,291	688	96
**4**	1.292	5,470	1,247	85
**5**	1.181	5,089	301	125
**6**	1.237	5,263	415	55
**7**	1.309	5,761	859	118
**8**	1.264	5,677	838	99
**9**	1.290	5,470	875	112
**10**	1.448	6,390	601	206
**11**	1.353	5,948	597	160
**12**	1.366	6,055	632	186
**13**	1.332	5,908	631	192
**14**	1.441	6,710	713	180
**15**	1.351	5,983	581	158
**16**	1.422	6,225	995	179
**17**	1.434	6,333	949	217
**18**	1.239	5,450	988	140
**19**	1.335	5,964	1,550	137
**20**	1.344	5,845	1,096	141
**21**	1.410	6,245	777	158
**22**	1.358	5,962	587	152
**23**	1.289	5,679	764	120
**24**	1.383	6,168	772	95
**25**	1.299	5,682	783	80
**26**	1.288	5,742	705	138
**27**	1.299	5,683	1,201	153
**28**	1.424	6,397	1,328	201
**29**	1.415	6,274	1,124	166
**30**	1.415	6,452	827	189

DM, dry matter; DMI, dry matter intake; GE, gross energy.

### Difference and correlation between DE, ME, and IVDE of feed ingredients

As shown in Table 5, the mean IVDE was 3,538 kcal/kg of DM across 20 feed ingredients, ranging from 1,910 to 4,975 kcal/kg of DM. The mean DE was 3,723 kcal/kg of DM, ranging from 1,774 to 5,135 kcal/kg of DM, while the mean ME was 3,563 kcal/kg of DM, ranging from 1,638 to 4,906 kcal/kg of DM. The mean IVDE: DE was 0.95, ranging from 0.84 to 1.08. Among these feed ingredients, the IVDE: DE of soybean meal 1, soybean meal 2, soy protein concentrate, rapeseed meal, and wheat bran were less than 0.90, while others ranged between 0.91 and 1.08. Notably, corn, wheat, and defatted rice bran exhibited an IVDE: DE of 1.00. Interestingly, the mean IVDE: ME was 0.99. However, the IVDE: ME of soy protein concentrate, sunflower meal, and rice DDGS deviated significantly from 1.00, while the ratios for other ingredients ranged between 0.90 and 1.06. The CV for the determined IVDE was lower than that of *in vivo* determined DE and ME, ranging from 0.12% to 1.37%, 0.68% to 6.54%, and 0.66% to 9.50%, respectively.

**Table 5:** The energetic values of 20 feed ingredients determined in vitro and in vivo (kcal/kg, DM basis).

Item	In vitro		In vivo				IVDE: DE	IVDE: ME	Predicted values			
	IVDE	CV, %	DE	CV, %	ME	CV, %			DE	CI	ME	CI
**Corn**	3,969	0.12	3,986	1.13	3,892	1.35	1.00	1.02	4,155	3,984–4,326	4,001	3,860–4,141
**Wheat**	3,867	0.30	3,854	1.56	3,777	2.22	1.00	1.02	4,053	3,892–4,214	3,897	3,765–4,029
**Sorghum**	4,012	0.28	3,886	1.10	3,802	1.38	1.03	1.06	4,198	4,022–4,374	4,044	3,899–4,189
**Barley**	3,337	0.56	3,363	2.23	3,295	2.34	0.99	1.01	3,522	3,370–3,673	3,359	3,234–3,483
**Wheat flour**	4,197	0.19	4,159	1.10	4,050	0.86	1.01	1.04	4,383	4,182–4,584	4,232	4,067–4,397
**Brown rice**	4,156	0.19	4,034	0.68	3,987	0.66	1.03	1.04	4,342	4,147–4,537	4,190	4,030–4,351
**Wheat middling**	3,452	0.39	3,748	2.88	3,655	1.85	0.92	0.94	3,637	3,491–3,784	3,476	3,355–3,596
**Soybean meal 1**	3,709	0.44	4,377	2.67	4,110	3.4	0.85	0.90	3,894	3,745–4,044	3,737	3,613–3,860
**Soybean meal 2**	3,734	0.47	4,245	3.02	4,064	4.07	0.88	0.92	3,920	3,768–4,071	3,762	3,638–3,886
**Fermented soybean meal**	3,627	0.31	4,260	2.06	4,019	2.24	0.85	0.90	3,812	3,666–3,959	3,653	3,533–3,774
**Enzyme-hydrolyzed soybean meal**	3,872	0.32	4,271	4.21	3,983	6.86	0.91	0.97	4,058	3,896–4,219	3,902	3,769–4,035
**Extruded soybean**	4,975	0.14	5,135	4.09	4,906	1.94	0.97	1.01	5,162	4,827–5,497	5,022	4,747–5,297
**Soy protein concentrate**	3,822	0.20	4,556	1.03	4,336	3.96	0.84	0.88	4,008	3,850–4,165	3,851	3,722–3,980
**Cottonseed meal**	2,861	0.72	2,996	2.24	2,725	8.27	0.95	1.05	3,045	2,842–3,249	2,876	2,708–3,043
**Rapeseed meal**	2,946	0.35	3,440	5.41	3,110	7.55	0.86	0.95	3,130	2,939–3,322	2,962	2,805–3,119
**Sunflower meal**	2,954	0.77	2,846	6.54	2,650	9.50	1.04	1.11	3,138	2,948–3,329	2,970	2,814–3,126
**Rice DDGS**	1,910	1.37	1,774	4.46	1,638	3.60	1.08	1.17	2,093	1,722–2,464	1,910	1,605–2,215
**Rice bran**	4,076	0.54	3,891	4.20	3,842	2.88	1.05	1.06	4262	4,078–4,446	4,109	3,958–4,261
**Defatted rice bran**	2,966	0.41	2,960	4.40	2,877	4.96	1.00	1.03	3,150	2,962–3,339	2,982	2,827–3,137
**Wheat bran**	2,320	0.64	2,686	4.67	2,543	4.25	0.86	0.91	2,504	2,209–2,798	2,326	2,085–2,568

CI, confidence intervals; CV, coefficient of variation; DDGS, distillers dried grains with solubles; DE, digestible energy; IVDE, in vitro digestible energy; ME, metabolizable energy.

**Figure 2:**
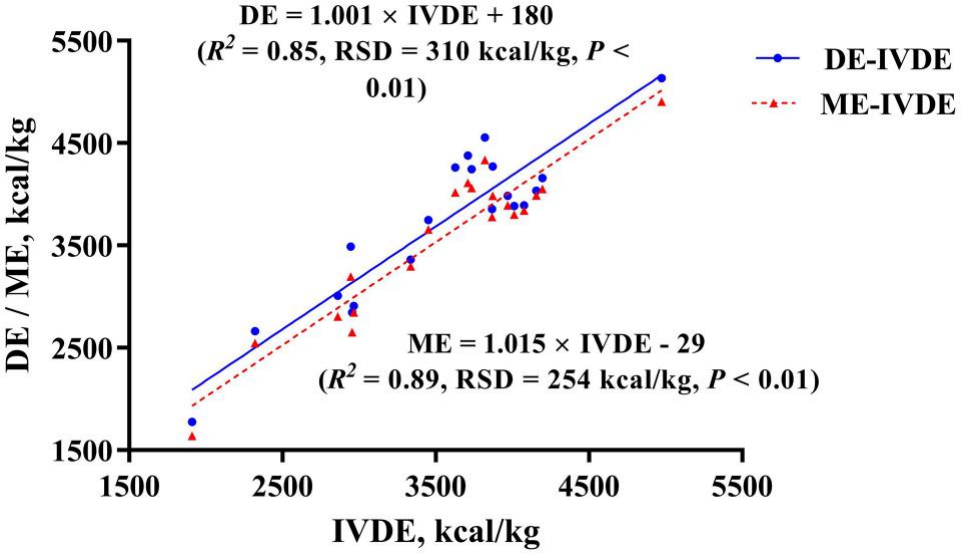
Prediction of DE and ME from IVDE of 20 feed ingredients. The DE and ME values were determined with 6 replicates of 1 growing pig per replicate for each sample, and IVDE values were determined based on the mean of 5 replicates per sample. DE, digestible energy; IVDE, *in vitro* digestible energy; ME, metabolizable energy; RSD, residual standard deviation.

The regression models for DE and ME against IVDE in 20 feed ingredients are presented in Figure 2: DE = 1.001 × IVDE + 180 (*R^2^* = 0.85, RSD = 310 kcal/kg, *P *< 0.01) and ME = 1.015 × IVDE—29 (*R^2^* = 0.89, RSD = 254 kcal/kg, *P *< 0.01), respectively. In these two models, the slopes (*P *= 0.88) and intercepts (*P *= 0.50) were not different, indicating a strong correlation (*r *= 0.99) between DE and ME. Calculated from these two prediction models, the predicted DE and ME of the 20 feed ingredients all fell within the 95% confidence intervals.

### Additivity of DE, ME, and IVDE in complete diets calculated from feed ingredients

**Figure 3:**
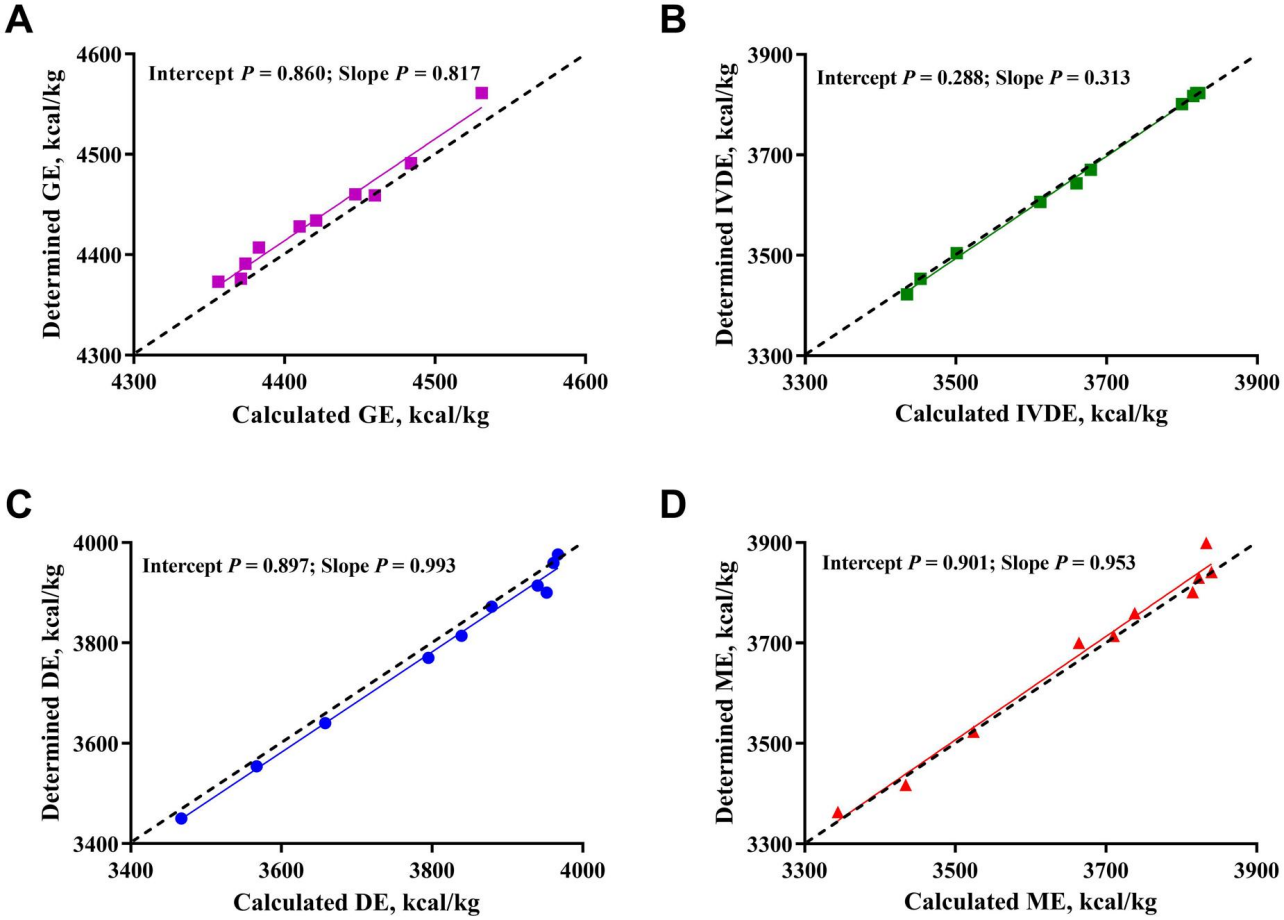
Regression analysis of the calculated and determined energetic values in 10 validation diets. A. analysis of GE values; B. analysis of IVDE values; C. analysis of DE values; D. analysis of ME values. DE, digestible energy; GE, gross energy; IVDE, *in vitro* digestible energy; ME, metabolizable energy.

Validation diets 1 to 10 were formulated with three to 6 of the 20 feed ingredients. The determined GE, IVDE, DE, and ME of the 10 validation diets ranged from 4,373 to 4,561, 3,422 to 3,823, 3,450 to 3,976, and 3,417 to 3,839 kcal/kg of DM, respectively (Table 6). The absolute difference between the determined and calculated GE was less than 20 kcal/kg of DM for eight diets and 20 to 30 kcal/kg of DM for two diets. The absolute differences between the determined and calculated values of IVDE, DE, and ME for the 10 validation diets ranged from 0 to 17, 2 to 52, and 0 to 33 kcal/kg of DM, respectively. There were no differences between the determined and calculated values of GE, IVDE, DE, and ME. Regression analysis of the calculated values against determined values is shown in Figure 3. The slopes of the regression models for GE, IVDE, DE, and ME were not different from 1, and the intercepts were not different from 0.

**Table 6:** The differences between the determined and calculated energetic values in 10 validation diets for growing pigs (kcal/kg, DM basis).

Diets	GE			IVDE			DE			ME		
	Determined	Calculated	Difference^1^	Determined	Calculated	Difference	Determined	Calculated	Difference	Determined	Calculated	Difference
**1**	4,428	4,410	18	3,643	3,660	−17	3,872	3,879	−7	3,759	3,738	21
**2**	4,391	4,374	17	3,817	3,815	2	3,959	3,961	−2	3,841	3,839	2
**3**	4,407	4,383	24	3,670	3,679	−9	3,814	3,839	−25	3,720	3,709	11
**4**	4,460	4,447	13	3,823	3,823	0	3,900	3,952	−52	3,830	3,829	1
**5**	4,373	4,356	17	3,606	3,612	−6	3,770	3,795	−25	3,700	3,667	33
**6**	4,459	4,460	−1	3,823	3,819	4	3,914	3,940	−26	3,801	3,821	−20
**7**	4,376	4,371	5	3,453	3,453	0	3,450	3,467	−17	3,331	3,335	−4
**8**	4,491	4,484	7	3,422	3,435	−13	3,554	3,567	−13	3,417	3,417	0
**9**	4,434	4,421	13	3,504	3,501	3	3,640	3,658	−18	3,523	3,507	16
**10**	4,561	4,531	30	3,801	3,800	1	3,976	3,967	9	3,842	3,833	9
**SEM**	18	18	–	50	49	–	57	57	–	59	59	–
***P* value**	0.940			0.959			0.829			0.935		

DE, digestible energy; GE, gross energy; IVDE, in vitro digestible energy; ME, metabolizable energy.

1 Difference is calculated by subtracting calculated energy from determined values.

### Accuracy of DE and ME predicted from IVDE

**Figure 4:**
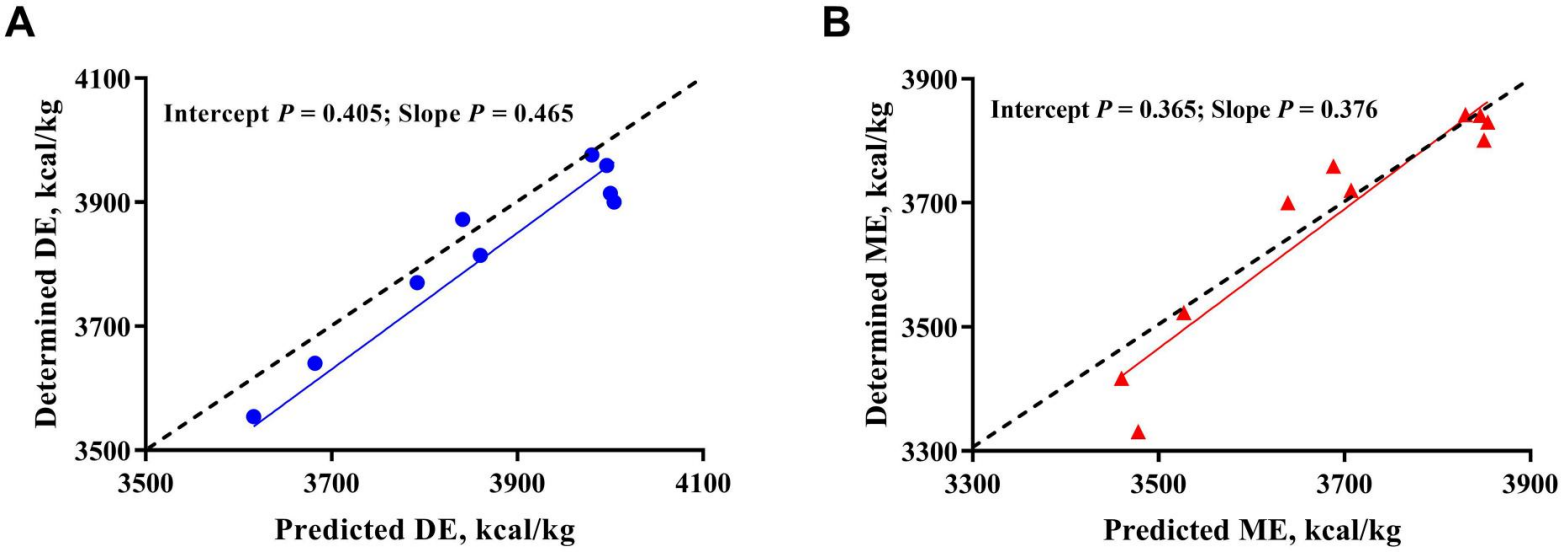
Regression analysis of the predicted and determined available energy in 10 validation diets. A. analysis of DE values; B. analysis of ME values. DE, digestible energy; ME, metabolizable energy.

Based on the proportion of feed ingredients in the validation diets and the predicted DE and ME derived from the equation in Figure 2, the predicted DE and ME are presented in Table 7. The determined and predicted DE values did not differ in eight out of 10 diets, with differences of less than 100 kcal/kg of DM. However, differences were observed in diets 4 and 7, with differences ranging from 100 to 200 kcal/kg of DM (*P *= 0.001 and < 0.001, respectively). For ME, values did not differ in nine out of 10 diets, with differences of less than 100 kcal/kg of DM. The exception was diet 7, which showed a difference (*P *< 0.001) of 147 kcal/kg of DM. Additionally, the single sample *t*-test results demonstrated that the determined DE values differed from the predicted values for diets 4, 6, and 7. In contrast, for ME, a difference between determined and predicted values were observed only for Diet 7. The absolute relative differences between the predicted and determined values of DE and ME were 2.08% and 1.62%, respectively. Regression analysis of the predicted values against the determined values showed that the slopes of the models for DE and ME were not different from 1, and the intercepts were not different from 0 (Figure 4).

**Table 7:** The differences between the determined and predicted available energy in 10 validation diets for growing pigs (kcal/kg, DM basis).

Diets	DE vs. IVDE					ME vs. IVDE				
	Determined	Predicted	Difference^1^	SEM	*P* value	Determined	Predicted	Difference	SEM	*P* value
**1**	3,872	3,841	31	25.3	0.275	3,759	3,688	71	33.2	0.086
**2**	3,959	3,996	−37	18.6	0.102	3,841	3,846	−5	40.7	0.919
**3**	3,814	3,860	−46	19.5	0.076	3,720	3,707	13	9.2	0.236
**4**	3,900	4,004	−104	15.6	0.001	3,830	3,854	−24	18.7	0.256
**5**	3,770	3,792	−22	13.9	0.181	3,700	3,639	61	25.3	0.061
**6**	3,914	4,000	−86	17.5	0.004	3,801	3,850	−49	22.1	0.077
**7**	3,450	3,633	−183	6.3	<0.001	3,331	3,478	−147	13.0	<0.001
**8**	3,554	3,616	−62	24.4	0.053	3,417	3,460	−43	19.3	0.077
**9**	3,640	3,682	−42	19.1	0.093	3,523	3,527	−4	22.4	0.880
**10**	3,976	3,980	−4	10.1	0.702	3,842	3,830	12	11.3	0.376
**SEM**	57	49				59	50			
***P* value**	0.470					0.883				
**ARD^2^, %**			2.08					1.62		

ARD, average relative difference; DE, digestible energy; IVDE, *in vitro* digestible energy; ME, metabolizable energy.

1 Difference was calculated by subtracting the predicted available energy from the determined value. Statistical significant is indicated at *P *< 0.05.

2 Calculated according to equation described in the Statistical Analysis.

## Discussion

In the current study, the 20 feed ingredients analyzed represent the most widely utilized components in Chinese pig production. A comparison of their GE values with the China National Standard of Nutrient Requirements of Swine (State Administration for Market Regulation, National Standardization Administration 2020) showed good agreement with most ingredients, with difference below 100 kcal/kg of DM. Notable exceptions were wheat flour, wheat middling, and rapeseed meal, which showed greater differences of 159, 202, and 123 kcal/kg of DM, respectively. Comparisons with the NRC (2012) database showed larger variations, with only nine out of 18 comparable ingredients differing by less than 100 kcal/kg of DM. More critically, the DE and ME of processed by-products, such as defatted rice bran, soybean meals, rapeseed meal, and wheat bran, which were deviated from CNRS reference values by more than 300 kcal/kg of DM. This substantial variability is intrinsic to processed by-products, arising from differences in source material, plant species, and processing methods, which collectively induce wide fluctuations in nutrient composition and resultant available energy (Stein et al. 2016). For example, reported DE and ME values vary considerably within ingredient categories: 2,039 to 3,157 and 1,931 to 2,978 kcal/kg of DM for nine defatted rice bran samples (Huang et al. 2018b), 3,889 to 4,419 and 3,803 to 4,257 kcal/kg of DM for 22 soybean meal samples (Li et al. 2015), and 3,143 to 4,252 and 2,816 to 3,933 kcal/kg of DM for 22 rapeseed meal samples (Maison et al. 2015), respectively. A key methodological factor contributing to database variance is the prevalent use of the substitution method for evaluating these ingredients, necessitated by their poor palatability or the inability to feed them as the sole dietary component for a sufficient duration to directly determine energy digestibility (Kong and Adeola 2014). This method, which formulates test diets by blending the test feed ingredient with a basal diet like corn, introduces cumulative errors. Du et al. (2022) reported that when the proportion of plant protein meals in the experimental diet was close to 20%, the mean CV of DE increased nearly six-fold, from 0.80% in diets to 4.84% in feed ingredients. Similarly, in this study, the mean CV of DE in six cereal grains was 1.30% when determined using the direct method, but it increased to 3.71% when the substitution method was used for other samples. These findings imply that the available energy of feed ingredients for growing pigs varied largely and did not meet the requirements of precise feed formulations when relying solely on static databases.

In vitro digestion method is applicable for predicting feed nutritive values due to its operability, time efficiency, and cost-effectiveness (Boisen and Fernández 1997; Woyengo et al. 2016; Sol et al. 2017). A strong relationship between in vitro and in vivo digestibility of GE was observed for 32 feed ingredients (*r *= 0.82; Bosien and Fernandez, 1997), 21 barley samples (*r *= 0.90; Regmi et al. 2008), and 20 wheat samples (*r *= 0.85; Regmi et al. 2009). However, the relative deviation between IVDE and in vivo determined values was greater than 10%, indicating that the static *in vitro* procedure inadequately simulated the in vivo physiology. Key limitations of static methods include the use of fixed digestion times, single pH points, and poorly defined enzyme cocktails (eg, pancreatin with high batch-to-batch activity variation). Crucially, they lack the dynamic, time-dependent coordination of pH changes, enzyme secretion, and digesta transit that characterizes the living gastrointestinal tract. The novel developed CCSDS addresses these shortcomings by automating a physiologically dynamic protocol. The system parameters, including temperature, pH, ion concentrations, and segment-specific digestion times, which were calibrated using in vivo data from growing pigs (Dang et al. 2018; Du et al. 2022, 2023; Gao et al. 2023). A significant advancement is the use of enzymes extracted and purified from porcine jejunal and cecal fluids, ensuring biological agreement with in vivo digestion. This dynamic approach demonstrated a high predictive accuracy, with the IVDE of the 20 feed ingredients showing correlation coefficients of 0.92 and 0.95 with in vivo DE and ME, respectively. The average IVDE: DE and IVDE: ME of 0.95 and 0.99 further confirm that the CCSDS closely approximates the overall in vivo energy release process.

Despite the strong overall correlations, systematic patterns in prediction accuracy were observed across ingredient types. For cereal grains (eg, corn, wheat), the IVDE was very close to DE, with a relative ratio approximately equal to 1.00. In contrast, for protein feed ingredients, like soybean meal, soy protein concentrate, and rapeseed meal, the IVDE: DE was below 0.90, indicating a consistent underestimation of available energy. This discrepancy is likely due to at least two model limitations. First, the formation of a gelatinous layer during the *in vitro* gastric digestion of proteins may impede the enzyme from digesting the substrate *in vitro*, a physical constraint less relevant *in vivo* where peristalsis and continuous enzyme secretion prevail (Sensoy 2021). Second, and more critically, is the simplified simulation of hindgut fermentation. The large intestine contributes over 20% of the energy digestibility for protein meals and high-fiber ingredients in pigs through microbial production of short-chain fatty acids (Woyengo et al. 2016; Huang et al. 2017b; Abelilla and Stein 2019). Our current CCSDS protocol, which uses purified enzymes to simulate large intestine digestion, accounts for less than 15% of this activity (Du et al. 2023), likely explaining the underestimation for relevant ingredients. While incorporating fresh fecal inocula is a common approach to simulate the colonic microbial community, its predictive accuracy can be limited. For instance, Huang et al. (2018a) observed a poor correlation (*r *≤ 0.70) between such in vitro fermentation and the in vivo energy digestibility of DDGS in growing pigs. This was mainly due to the high variability of microorganisms in feces and the challenges in standardizing the inoculum (Tao et al. 2019). More physiologically relevant approaches using cecal or colonic digesta as inoculum have been explored to better replicate the autochthonous microbiota (Montoya et al. 2021; Durif et al. 2025). However, these methods introduce practical complexities, hindering their adaptation for high-throughput applications like the one presented in this study. The high correlation coefficients (*r *> 0.90) confirm that the CCSDS reliably ranks ingredients based on their available energy, which is invaluable for comparative formulation. Nevertheless, a consistent underestimation for certain ingredient classes reveals a systematic bias. Biologically, this means that while the model excellently captures foregut enzymatic digestion, the microbial energy contribution from the hindgut is quantified with greater uncertainty. Practically, this implies that absolute ME predictions for high-fiber or protein-rich by-products should be applied with caution, potentially requiring calibration with *in vivo* data for specific ingredient batches.

Nutrient additivity is a fundamental assumption in feed formulation for pigs, which assumes that the nutrients in a mixed diet are equal to the proportional sum of those from the individual feed ingredients (Stein et al. 2005; Kim et al. 2017; Wang et al. 2022). Consequently, validating the additivity of energy is essential for assessing the accuracy of in vitro methods designed to determine the available energy of feed ingredients. The results clearly demonstrated the additivity of GE, IVDE, DE, and ME in the 10 mixed validation diets. No differences were observed between the determined and calculated values, and their linear relationship overlapped with the line of Y = X. Moreover, the absolute differences between determined and calculated values of GE, IVDE, DE, and ME were less than 30, 17, 52, and 33 kcal/kg of DM, respectively. These differences are well within the accepted error threshold of 100 kcal/kg of DM for swine energy bioassays (Kim et al. 2017), confirming the accuracy of the energy determinations for feed ingredients and mixed diets in this study. Analysis of the predicted DE and ME in validation diets further supports the system’s reliability. In 90.0% of validation diets (9/10 diet), the difference between determined and predicted available energy was less than 104 kcal/kg of DM. This performance compares favorably with the 82.3% report for a previous manual in vitro method (Boisen and Fernández 1997). A single exception was validation diet 7 (composed of corn, defatted rice bran, cottonseed meal, and sunflower meal), where the difference exceeded 100 kcal/kg DM. This discrepancy arose because the predicted DE and ME values for all four constituent ingredients were higher than the determined DE, leading to cumulative variation in the mixed diet prediction. A high correlation coefficient (*r *> 0.90) between the predicted and determined available energy across 10 validation diets indicates that variation in vivo is consistently reflected in vitro. Collectively, these results suggest that the CCSDS was more accurate than manual in vitro digestion for predicting the available energy of feed ingredients for growing pigs.

## Conclusion

This study demonstrates that the novel CCSDS provides a highly accurate and physiologically relevant prediction of available energy in feed ingredients, closely aligning with *in vivo* measurements for monogastric species. The CCSDS achieved strong correlations with *in vivo* measurements, with IVDE: DE and IVDE: ME approximating 0.95 and 0.99, respectively. Additionally, the high degree of energy additivity observed across validation diets confirms the reliability and consistency of the predicted values. These findings support the use of CCSDS as an efficient, high-throughput alternative to conventional in vivo assays, with significant potential for application in the formulation of optimized monogastric feeds.

## Data Availability

The data underlying this article will be shared on reasonable request to the corresponding author.

## Abbreviations

ADF: acid detergent fiber
ARD: average relative difference
BW: body weight
CCSDS: computer-controlled simulated digestion system
CI: confidence intervals
CNRS: China National Standard of Nutrient Requirements of Swine
CP: crude protein
CV: coefficient of variation
DDGS: distillers dried grains with solubles
DE: digestible energy
DM: dry matter
EE: ether extract
GE: gross energy
IVDE: in vitro digestible energy
ME: metabolizable energy
NDF: neutral detergent fiber
RSD: residual standard deviation
